# *Mycobacterium tuberculosis* Calcium Pump CtpF Modulates the Autophagosome in an mTOR-Dependent Manner

**DOI:** 10.3389/fcimb.2020.00461

**Published:** 2020-09-16

**Authors:** Rajni Garg, Salik Miskat Borbora, Harsh Bansia, Sandhya Rao, Prakruti Singh, Rinkee Verma, Kithiganahalli Narayanaswamy Balaji, Valakunja Nagaraja

**Affiliations:** ^1^Department of Microbiology and Cell Biology, Indian Institute of Science, Bangalore, India; ^2^Jawaharlal Nehru Centre for Advanced Scientific Research, Bangalore, India

**Keywords:** tuberculosis, *Mycobacterium tuberculosis*, CtpF, calcium, autophagy, mTOR

## Abstract

Calcium is a very important second messenger, whose concentration in various cellular compartments is under tight regulation. A disturbance in the levels of calcium in these compartments can play havoc in the cell, as it regulates various cellular processes by direct or indirect mechanisms. Here, we have investigated the functional importance of a calcium transporting P2A ATPase, CtpF of *Mycobacterium tuberculosis* (Mtb) in the pathogen's interaction with the host. Among its uncanny ways of dealing with the host with umpteen strategies for survival and persistence in humans, CtpF is identified as a new player. The levels of *ctpF* are upregulated in macrophage stresses like hypoxia, high nitric oxide levels and acidic pH. Using confocal microscopy and fluorimetry, we show that CtpF effluxes calcium in macrophages in early stages of Mtb infection. Downregulation of *ctpF* expression by conditional knockdown resulted in perturbation of host calcium levels and consequent decreased activation of mTOR. We present a mechanism how calcium efflux by the pathogen inhibits mTOR-dependent autophagy and enhances bacterial survival. Our work highlights how Mtb engages its metal efflux pumps to exploit host autophagic process for its proliferation.

## Introduction

Tuberculosis continues to be a serious concern to mankind. The phenomenon of dormancy associated with *Mycobacterium tuberculosis* (Mtb) fuels successful survival in the host (Gengenbacher and Kaufmann, 2012). Hypoxia, high levels of nitric oxide (NO) and carbon monoxide (CO) are signals for DevS (DosS) and DosT, the dormancy sensor kinases, which get phosphorylated and transmit the signal to DevR (DosR) response regulator (Voskuil et al., 2003; Saini et al., 2004; Kumar et al., 2008). The 48 genes which are under the control of DevRS two- component system, are upregulated under these conditions (Voskuil et al., 2003; Kumar et al., 2008). The DevRS regulon is highly expressed upon macrophage infection, suggesting that these genes might play a critical role in persistence/survival mechanisms of the bacterium (Voskuil and Schlesinger, 2015). While the functions of some of these genes have been studied, the functions of others are predicted or unknown.

Rv1997 or cation transporting P-type ATPase F (*ctpF)* is one such gene, which in addition to the regular cues of dormancy (Park et al., 2003; Voskuil et al., 2003; Kumar et al., 2008), is also upregulated in early infection, non-replicating persistence-1 (NRP-1) and NRP-2 states (Muttucumaru et al., 2004). It is one of the twelve P-type ATPases, detected in the proteome of Mtb H37Rv (Botella et al., 2011; Novoa-Aponte et al., 2012). P-type ATPases are integral membrane proteins, present in almost all domains of life and maintain active ion gradients across the cell membrane at the expense of ATP. The catalytic cycle of these ion pumps has been illustrated by the studies on SERCA1 (sarco/endoplasmic reticulum Ca^2+^-ATPase of rabbit muscle cells) ATPases (Olesen et al., 2004, 2007; Sorensen et al., 2004; Toyoshima and Mizutani, 2004). They oscillate between two conformational regiments: the metal bound form (E1) and the unbound form (E2) (Bublitz et al., 2011).

Previous studies have indicated the importance of the ionic milieu of Mtb phagosomes in influencing the activity of mycobacterial P-type ATPases. Mtb phagosomes have high concentration of heavy metals, leading to the induction of CtpC which is implicated in efflux of Zn^2+^ or Mn^2+^ (Botella et al., 2011; Padilla-Benavides et al., 2013). Similarly, CtpV which exports Cu^2+^, enables bacterial survival in such conditions (Ward et al., 2010; Wolschendorf et al., 2011). An unusual ATPase, CtpE is shown to be involved in uptake of Ca^2+^ ions by *M. smegmatis* (Gupta et al., 2017). Being a P2A ATPase, CtpF is predicted to be involved in alkali/alkaline earth metal transport (Novoa-Aponte et al., 2012). Here, we have characterized CtpF as a Ca^2+^-transporting P2A ATPase. Conditional knockdown (CKD) of *ctpF* lead to impaired mycobacterial survival in macrophages. Also, we show how CtpF contributes in autophagy inhibition, facilitating Mtb survival, by perturbing Ca^2+^ homoeostasis or levels in the cells.

## Results

### Reduction in *ctpF* levels Leads to Decreased Mycobacterial Survival in THP-1 Macrophages

**Figure 1:**
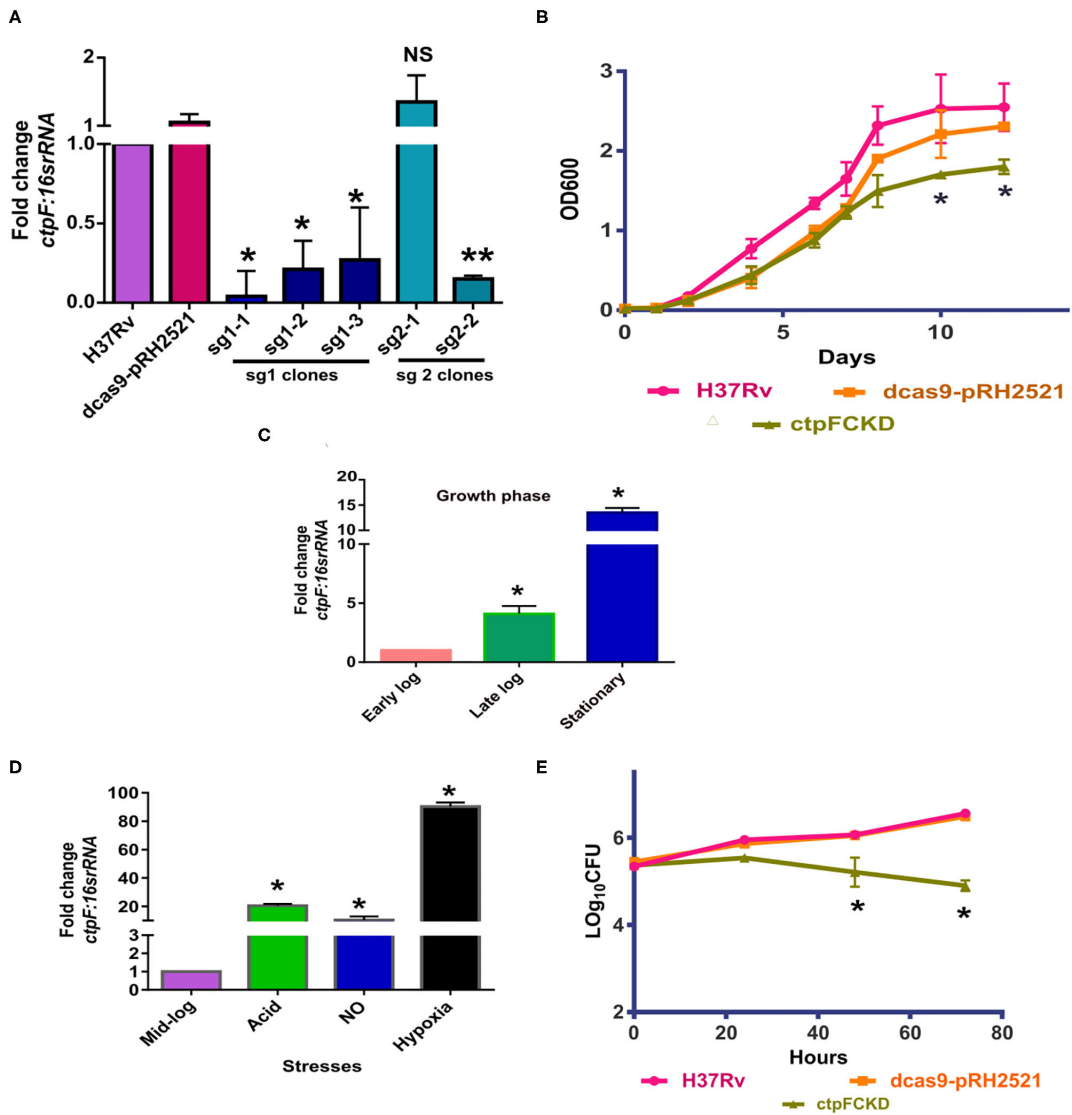
CtpFCKD strain is attenuated for growth in macrophages. **(A)** Screening of CtpFCKD colonies. Quantitative RT-PCR showing downregulation of *ctpF* transcript with respect to levels in Mtb H37Rv, in putative knockdown colonies with sgRNA1, and sgRNA2. **(B)** *In vitro* growth curve of Mtb H37Rv (pink), dcas9-pRH2521 (orange), and ctpFCKD (olive green) in 7H9-ADC media. Graph depicts fold change in *ctpF* transcript levels in Mtb H37Rv in late log and stationary phase as compared to early log phase **(C)** and in different stresses as compared to mid-log phase **(D)**. 16s rRNA was used as internal control in **(A,C,D)**. **(E)** Growth profile of Mtb H37Rv, dcas9-pRH2521, and ctpFCKD in THP-1 macrophages. Six hundred ng/ml ATc was added after every 48 h to maintain conditional knockdown in **(A,B)**. The values shown are mean of three independent experiments and error bars represent standard deviation. *, ** and NS indicate *p* < 0.05; *p* < 0.01 and *p-*value Non-significant, respectively, as analyzed by unpaired student's *t-*test.

Although *ctpF* is known to be a non-essential gene for *in vitro* growth of Mtb (Sassetti et al., 2003), its expression is upregulated in early stages of infection (Botella et al., 2011), suggesting its participation in intracellular survival of the organism. To investigate its role in Mtb physiology, before and after the infection, we resorted to down-regulate its expression. The conditional knockdown of *ctpF* (ctpFCKD) was generated in Mtb H37Rv deploying CRISPR-cas9 strategy, as described in the Materials and Methods section. Figure 1A shows *ctpF* transcript levels in putative knockdown colonies obtained using sgRNA1 and sgRNA2. The clone sg1-1 showing 25-fold reduction in *ctpF* expression was used as ctpFCKD for further experiments. Although in exponential phase cultures, no significant difference was seen in the *in vitro* growth of ctpFCKD as compared to Mtb H37Rv or dcas9-pRH2521 (vector control), substantial growth difference was observed during the stationary phase (Figure 1B). The 13-fold upregulation of *ctpF* transcript in stationary phase, as compared to early log phase in Mtb also suggested that CtpF confers growth advantage to Mtb in stationary phase (Figures 1B,C). *ctpF* transcript was also upregulated when exponential phase Mtb cultures were exposed to stress conditions, akin to those normally found in macrophages. The transcript levels increased by 5, 20, and 100-fold in presence of NO, acidic media and hypoxia, as compared to the untreated (Figure 1D). These results suggested that CtpF may have a more important role for Mtb survival intracellularly unlike aerobic *in vitro* growth. After 72 h of infection in THP-1 macrophages, Mtb and the vector control strains showed 1.26 and 1.05 log increase, respectively, from their initial CFUs, while the ctpFCKD CFUs did not show any increase (Figure 1E), indicating its role in intracellular survival of Mtb. Recovery of similar number of bacterial CFUs for Mtb, vector control and the knockdown at 0 h post infection, ruled out the possibility of initial uptake difference.

### Growth Defect of MsCtpF is Rescued by Ca^2+^ Supplementation

**Figure 2:**
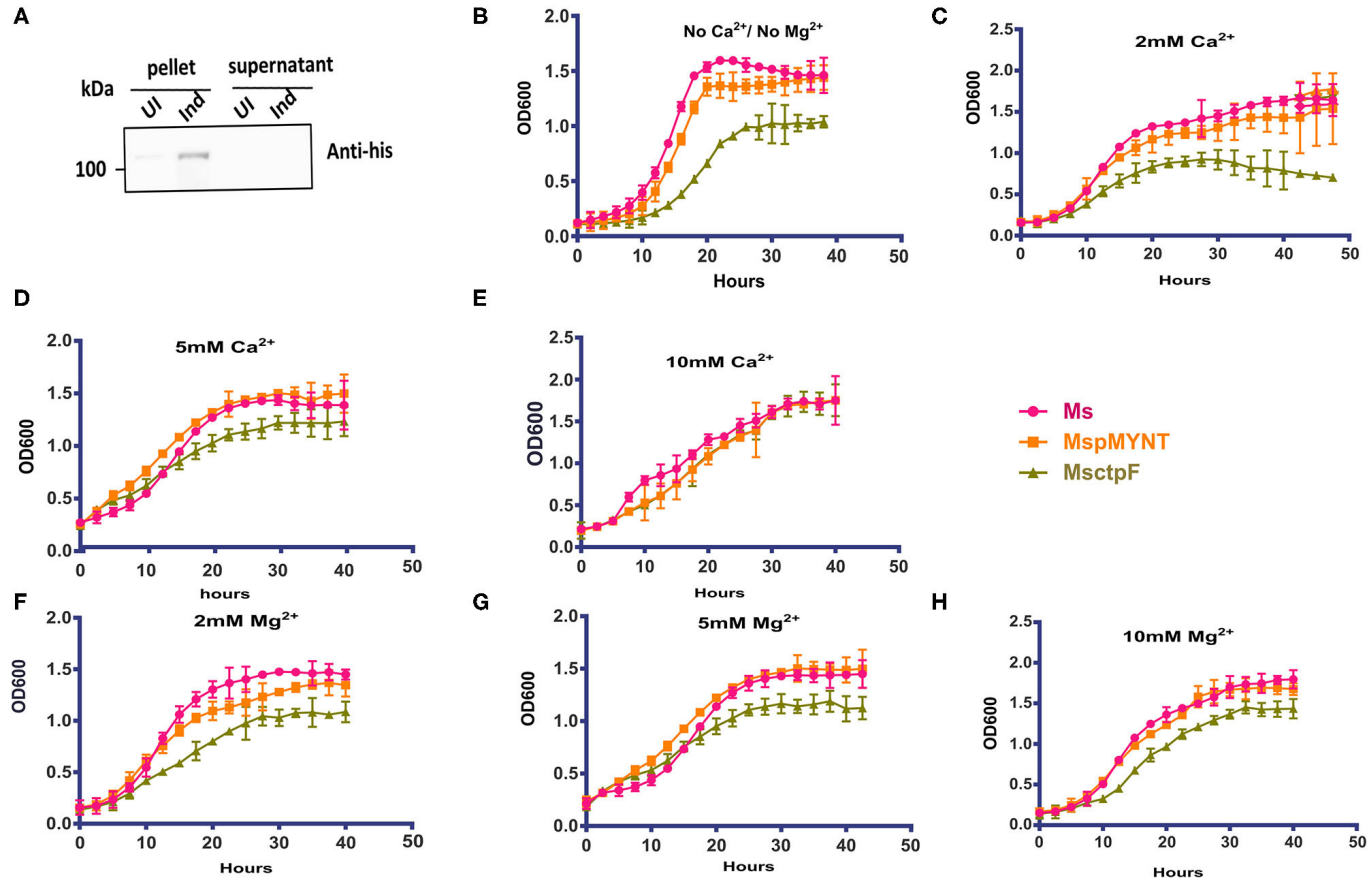
Growth defect of MsCtpF is rescued by Ca^2+^ ions. **(A)** Immunoblot showing hexa-histidine tagged CtpF expression in MsCtpF, post-induction, using anti-His antibody. Lanes representing the pellet and supernatant fractions of cultures uninduced (UI) and induced (Ind) with 2% acetamide are indicated. Growth curve of induced Ms (pink), MspMyNT (orange), and MsCtpF (olive green) in 7H9 media only **(B)**, with 2, 5, and 10 mM CaCl_2_ supplementation **(C–E)** and with 2, 5, and 10 mM MgCl_2_ **(F–H)**, respectively. The results shown are mean +/– S.D. from three independent experiments.

In a complementary approach to decipher the role of *ctpF, M. smegmatis* strain expressing hexa-histidine tagged CtpF (MsCtpF) was generated. The protein expression was confirmed by immunoblotting using anti-His antibody (Figure 2A). MsCtpF strain showed retarded growth in 7H9 medium, suggesting the possibility that excess metal efflux, could be depriving bacterium of metal needed for its physiological processes (Figure 2B). To determine whether the growth defect of MsCtpF gets rescued by externally provided metal ions, growth of *M. smegmatis* (Ms), MspMyNT (vector control), and MsCtpF strains was carried out with or without Ca^2+^ /Mg^2+^ ions. With the increase in Ca^2+^ concentration, increased rescue of growth was seen (Figures 2C–E). With 10 mM Ca^2+^ concentration, MsCtpF showed almost similar growth pattern as the vector control (Figure 2E). No rescue was observed in case of Mg^2+^ supplementation (Figures 2F–H), indicating that CtpF is likely to be a Ca^2+^ specific efflux pump.

### CtpF is a Bonafide Calcium ATPase

**Figure 3:**
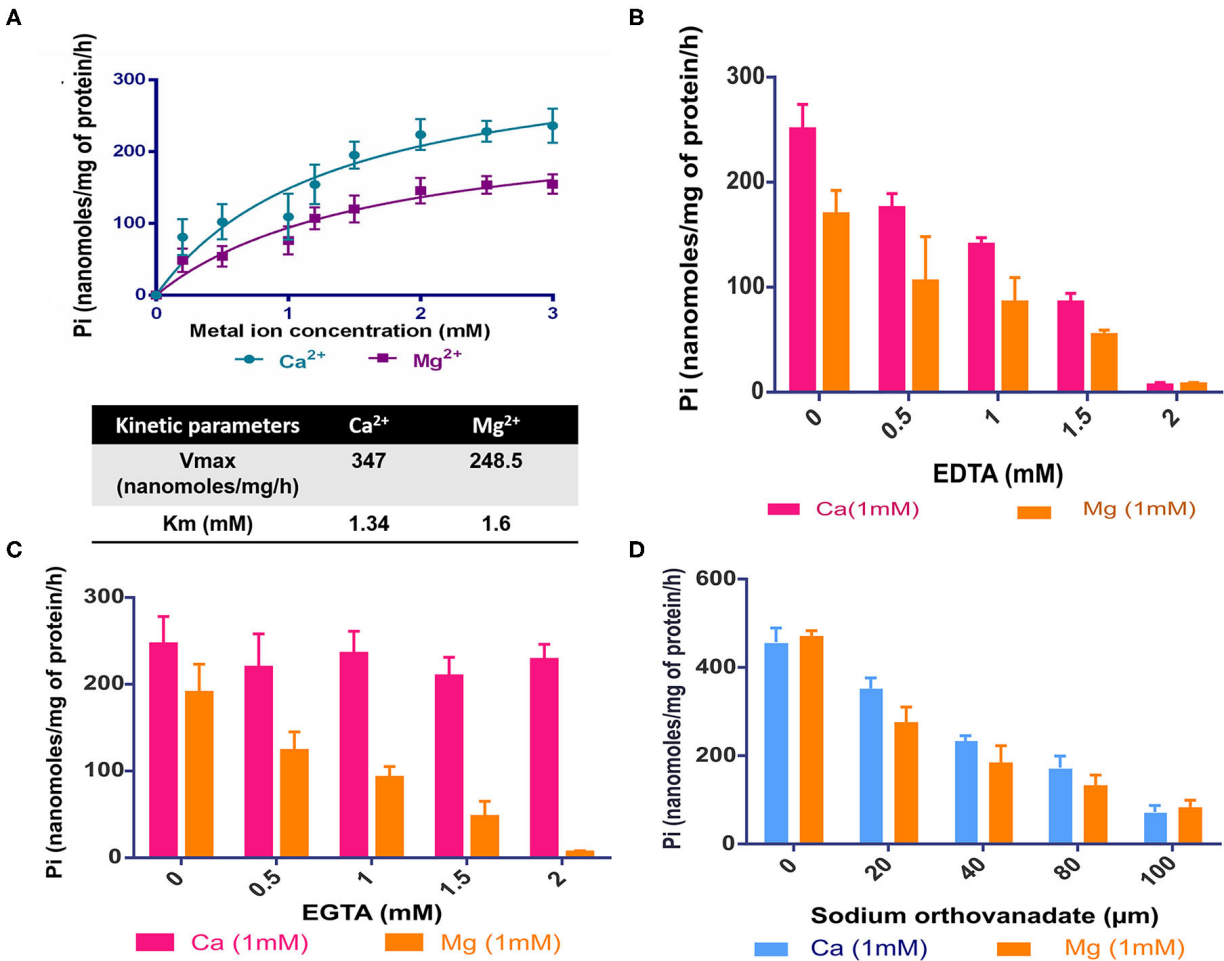
*In vitro* activity of CtpF **(A)** ATPase assay. Michaelis Menten curve showing nanomoles of Pi/mg of protein/h obtained with membrane vesicles of MsCtpF when stimulated with Ca^2+^ and Mg^2+^ ions. The absorbance values obtained by Malachite Green Assay were normalized with the only vector and no metal ion controls. The line curve with circular symbols show activity with Ca^2+^, while with square symbols show activity with Mg^2+^. K^+^ Inhibition of ATPase activity in presence of EDTA **(B)**, EGTA **(C)**, and sodium orthovanadate **(D)**.

Given its requirement for intracellular growth of Mtb, we sought to understand the biochemical properties of CtpF. To assess its *in vitro* activity, we adopted a strategy of isolation of membrane vesicles harboring CtpF, as it is an integral membrane protein. The ATPase activity of the protein is dependent on divalent metal ions. With increasing concentration of Ca^2+^ and Mg^2+^, increase in ATPase activity was observed (Figure 3A). No increase in the ATPase activity of CtpF vesicles was detected with monovalent ions: K^+^ and Na^+^. The ATPase activity of MsCtpF vesicles showed higher Vmax and lower Km with Ca^2+^ (347 nanomoles/mg protein/h and 1.34 mM, respectively) than Mg^2+^ ions (248.5 nanomoles/mg protein/h and 1.6 mM, respectively) (Figure 3A). Increasing concentrations of EDTA inhibited both Mg^2+^/Ca^2+^ mediated ATPase activity with maximum inhibition at 2 mM concentration, while EGTA only inhibited Ca^2+^ stimulated activity (Figures 3B,C). When sodium orthovanadate, a competitive inhibitor of ATPase activity was used in the reaction with both the ions, complete inhibition of Pi release was seen (Figure 3D). These results confirm the recent observations (Maya-Hoyos et al., 2019). Although the activity is seen with Mg^2+^ addition, albeit lower than Ca^2+^, it appears that *in vivo* Mg^2+^ is not the preferred substrate (Figure 2). Taking together, the results of growth rescue by Ca^2+^ supplementation and not by Mg^2+^ (previous section, Figure 2) and the Ca^2+^ dependent ATPase activity, we conclude that CtpF is a bonafide Ca^2+^ ATPase.

Modeled structure of CtpF using Ca^2+^ -bound SERCA1a in E1 confirmation (PDB code 1SU4) as the template is shown in Figure S1. Model of CtpF in an alternate confirmation E2 (with inhibitor Cyclopiazonic Acid bound) was reported recently (Santos et al., 2020). Although our results, in general, are in broad agreement with theirs, there are a few subtle differences. In their CtpF model, the drug Cyclopiazonic Acid (CPA) is docked onto the enzyme, in what appears to be a locked state. In support, their results also show inhibition of ATPase activity of the enzyme in presence of the drug (See discussion). CtpF has highest similarity (60.1%) as well as identity (33.3%) with SERCA1a, covering 99% query length. Like SERCA1a, the cytoplasmic region of CtpF has three domains viz Domain A, actuator or anchor; Domain N, nucleotide; and Domain P, phosphorylation, while membrane-localized region “M” contains two domains: domain T, transport and domain S, substrate-specific (Figures S1A,B). When the Ca^2+^ binding residues in SERCA1a structure (Clarke et al., 1989; Toyoshima et al., 2000), were superposed with the structurally equivalent residues in CtpF, except for D800 and A305, all the other Ca^2+^ binding residues were found to be conserved in both the proteins (Figures S1C,D). The functional residues inferred from structural and biochemical studies of SERCA were mapped onto the multiple sequence alignment (MSA) of CtpF orthologs across MTB complex (MTBC) and a few representative actinomycetes (Figure S2A). A cent percent conservation in ATP binding and phosphorylation residues was seen in all the test species. The Ca^2+^ binding residues were conserved in MTBC, but showed variation in *M. smegmatis, Streptomyces* and *Brevibacterium* sp. (Figure S2A). MSA of CtpF with Ca^2+^ ATPases from rabbit, pig, human and bovine, also showed conservation of ATP-binding, Ca^2+^ binding residues and the phosphorylation residue (Figure S2B). The modeling and MSA results reveal that the functional residues in CtpF (or its homologs) are conserved across diverse species.

### MsCtpF Shows Defective Biofilm, Pellicle, and Sliding Motility

From the previous sections, it is apparent that optimum level of *ctpF* expression is needed for mycobacterial growth. At lower level of expression of *ctpF*, intracellular growth of Mtb is affected, while its overexpression in *M. smegmatis* affected the *in vitro* growth. Hence, other phenotypic characteristics of MsCtpF strain were examined. A thin and fragile biofilm was observed for MsCtpF cultures (Figure S3A upper panel). The middle panel shows restoration of biofilm forming capacity of MsCtpF upon addition of 2 mM CaCl_2_, but not with same concentration of Mg^2+^ ions (Figure S3A, lower panel). MsCtpF also exhibited thin pellicle and reduced sliding motility which gets partially rescued by Ca^2+^ supplementation (Figures S3B,C). Decrease in biofilm in MsCtpF cultures, might be due to less availability of calcium for initial attachment and maturation of biofilm (Wang et al., 2019).

### CtpF Effluxes Calcium in Host Macrophages

**Figure 4:**
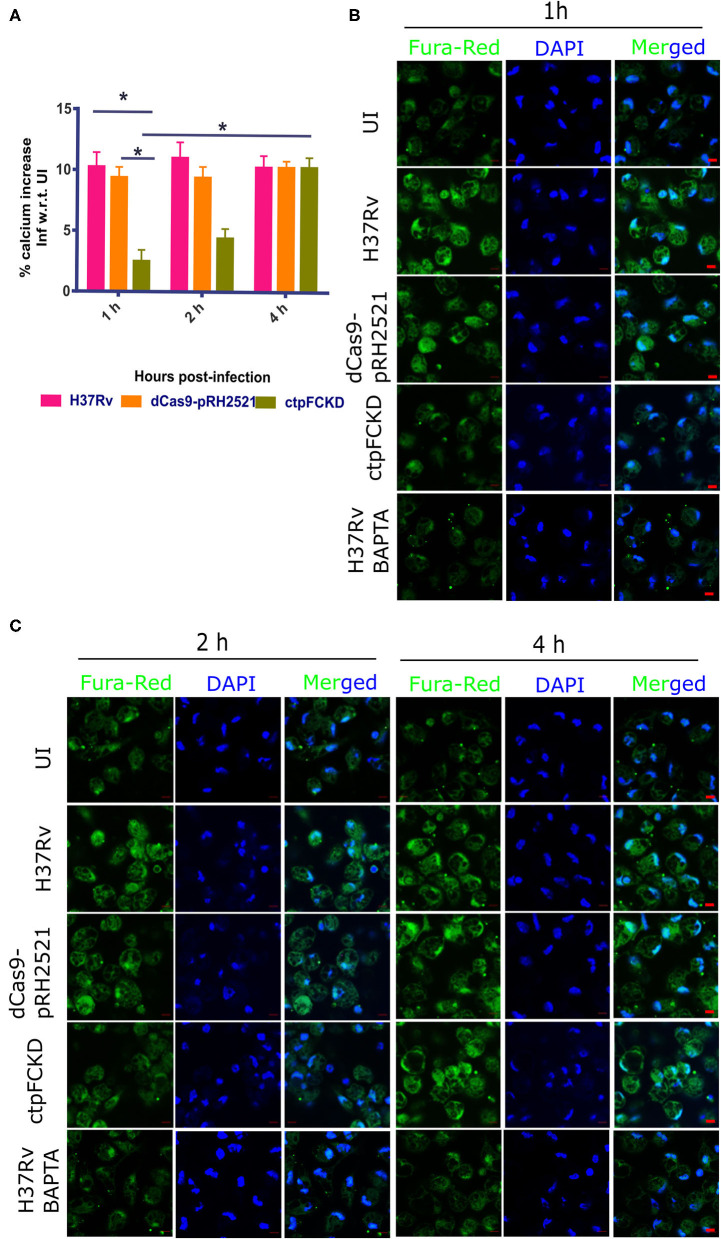
CtpF mediates calcium efflux into the host macrophages. **(A)** Percentage increase in calcium levels in mouse peritoneal macrophages after infection with Mtb H37Rv, dcas9-pRH2521, and ctpFCKD strains with respect to uninfected (UI) cells, at indicated time points. *Indicates *p* < 0.05, as calculated by unpaired student's *t*-test. Representative confocal images of macrophages loaded with Fura red, AM (pseudo-colored green), taken under 63X objective lens using 488 and 405 nm lasers (DAPI) at 1 h **(B)**, 2 h and 4 h **(C)** post-infection. Scale bars represent 5 μm.

Integrating the functionality of CtpF as a Ca^2+^ ATPase (this study) and elevated transcriptional levels of *ctpF* in the early stages of Mtb infection (Botella et al., 2011), we investigated whether CtpF is engaged in calcium efflux in host macrophages. Using ratiometric fluorimetry, the levels of calcium were measured *ex vivo*, in mouse peritoneal macrophages after infection with H37Rv, vector control and ctpFCKD. The total Ca^2+^ levels in macrophages infected with H37Rv and the vector control increased by 10.25 and 9.4%, 1 h post infection, respectively, as compared to the uninfected macrophages. Nearly same levels were maintained till 4 h post-infection in macrophages infected with both the strains (Figure 4A). No increase in calcium levels was seen in ctpFCKD, after 1 and 2 h of infection as compared to uninfected control. At 4 h post-infection, the levels of calcium in ctpFCKD were restored back to the levels, seen in macrophages infected with either WT or the vector control. In a separate experiment, at same time points, the infected macrophages were imaged by confocal microscopy. Figures 4B,C show representative confocal images of macrophages, post-infection with all the three strains. The infected macrophages treated with calcium chelator BAPTA, AM (last lanes of Figures 4B,C) were used as positive control and as expected they showed less Fura Red signal. No surge in Fura Red signal was seen post-infection with ctpFCKD in macrophages, indicating that CtpF mediates calcium efflux by Mtb at early time points after infection.

### CtpF-Mediated Ca^2+^ Efflux Leads to mTOR-Dependent Autophagy Inhibition in Macrophages

**Figure 5:**
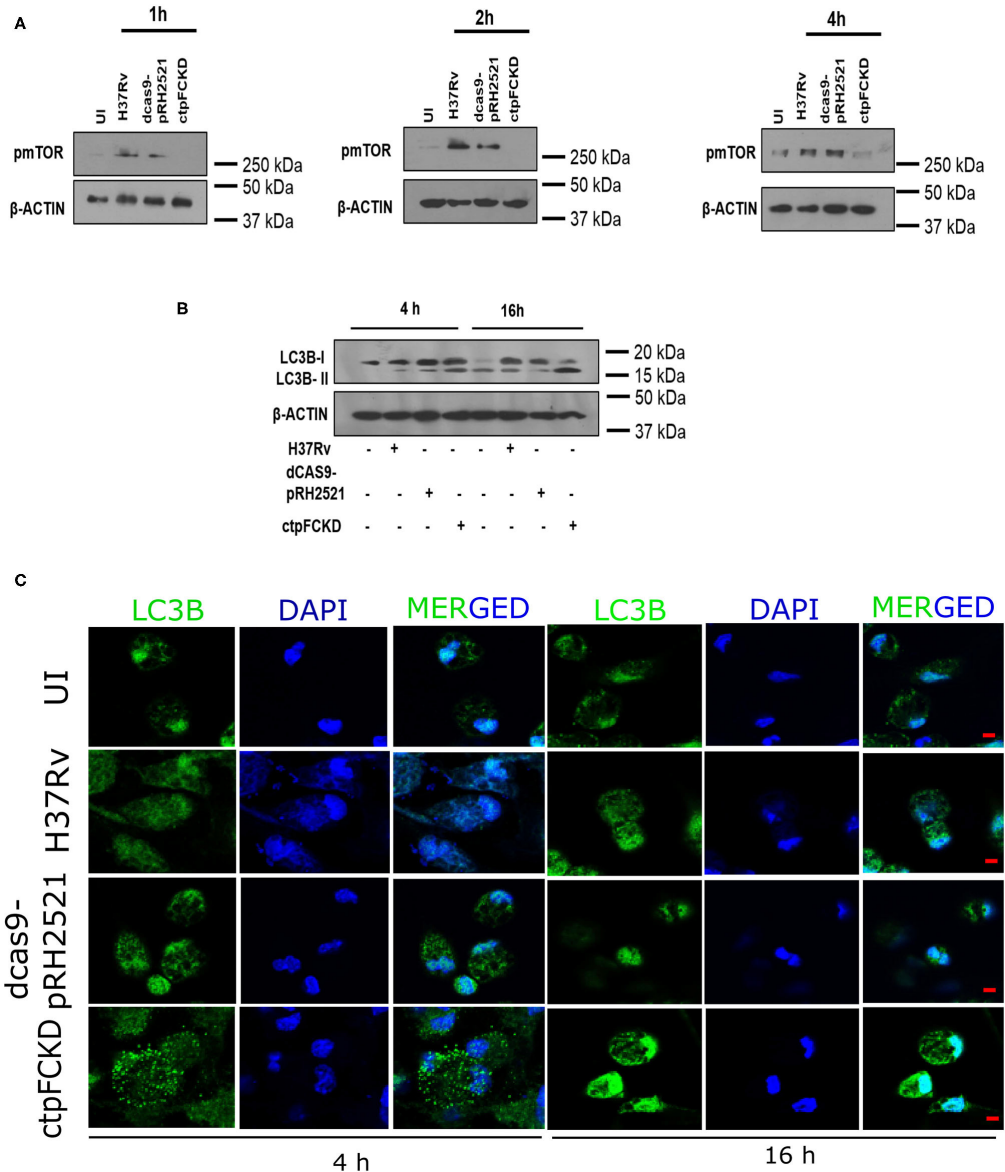
CtpF mediated Ca^2+^ efflux leads to mTOR-dependent autophagy inhibition in macrophages. Immunoblots showing decrease in p-mTOR **(A)** and conversion of LC3B-I to LC3B-II in uninfected (UI) and peritoneal macrophages infected with Mtb H37Rv, dcas9-pRH2521, and ctpFCKD strains at indicated time points **(B)**. Confocal images showing LC3B puncta at indicated time points after *ex vivo* infection with Mtb H37Rv, dcas9-pRH2521, ctpFCKD strains, and uninfected cells (UI) **(C)**. Scale bars represent 5 μm.

From the experiments presented above, it is evident that CtpF is a player in Ca^2+^ efflux from pathogen to the host, which may trigger further downstream events. Calcium flux regulates various events such as apoptosis, inflammasome regulation, necroptotic cell death etc. in the host (Harr and Distelhorst, 2010). Signaling mechanisms such as mammalian target of rapamycin (mTOR) pathway are also regulated by calcium levels in the cell. mTOR is a serine/threonine kinase and master regulator of autophagy in the cell (Gutierrez et al., 2004). Previous reports suggest activation of mTOR, as one of the mechanisms for inhibiting autophagy by Mtb to ensure its survival in the host (Gutierrez et al., 2004; Jo, 2013). In murine peritoneal macrophages, Mtb H37Rv infection resulted in an increase in mTOR phosphorylation (Figure 5A). The p-mTOR was nearly absent at 1 and 2 h post-infection with ctpFCKD, as opposed to H37Rv or the vector control. However, after 4 h of infection, p-mTOR signal could be detected in the ctpFCKD, suggesting the gradual restoration of p-mTOR levels. These results corroborate with the restored calcium levels at 4 h, presented in the above section (Figure 4A), indicating how mycobacterial calcium efflux may regulate phosphorylation of mTOR. To ascertain whether reduced p-mTOR level is responsible for enhanced clearance of ctpFCKD (Figure 1E), induction of autophagy was examined. Macrophages infected with ctpFCKD showed increased conversion of microtubule-associated protein 1A/1B-light chain 3B I (LC3B-I) to the lipidated autophagosome membrane marker LC3B-II at 4 and 16 h post-infection, as compared to uninfected or macrophages infected with WT or the vector control (Figure 5B). At 16 h post infection, most of LC3B-I in ctpFCKD infected macrophages is converted to LC3B-II, suggesting autophagy-dependent killing of ctpFCKD. Higher number of LC3B puncta observed by confocal microscopy, after 16 h of infection with CtpFCKD also suggested autophagy induction (Figure 5C).

## Discussion

Interplay of Mtb with the host involves multiple facets and the homoeostasis of micronutrients appear to be one of them (Ward et al., 2010; MacGilvary et al., 2019). Thus, the fine tuning of the expression of P-type ATPases which maintain metal ion gradients of the organism across the membrane would ensure Mtb to cope up with excess or scarcity of metal ions, during infection. Indeed, transcriptomic studies showed upregulation of *ctpG, ctpC, ctpF*, and *ctpV* and downregulation of *ctpD, ctpE*, and *ctpA* in intracellular mycobacteria (Botella et al., 2011). Each one of the P-type ATPases that are differentially expressed upon Mtb infection, partakes in different metal ion transport highlighting the requirement for their traffic across the mycobacterial membrane. For example, Mtb takes care of heavy metal quota of phagosomes through export of zinc and copper ions, via CtpC and CtpV, respectively (Ward et al., 2010; Botella et al., 2011; Wolschendorf et al., 2011). While CtpC is essential for virulence in mice and guinea pigs, CtpV mutant has defective pathology in mice, with no reduction in bacterial burden (Ward et al., 2010; Padilla-Benavides et al., 2013). CtpC is also involved in transport of Mn^2+^ and in secreted protein metalation (Padilla-Benavides et al., 2013). Calcium being a major second messenger, is one of the key players in physiology of both host and diverse pathogens (Rosch et al., 2008; Faxén et al., 2011). While calcium has an established role in phagosome biogenesis, which is a crucial determinant in Mtb pathogenesis (Vaeth et al., 2015), the levels of calcium in Mtb phagosomes or effects of its perturbation has not been understood. The role of CtpF described here in controlling the calcium transport across the membrane provides some insight in this aspect. The upregulation of *ctpF* and downregulation of Ca^2+^ influx pump *ctpE* upon Mtb infection (Botella et al., 2011), appears to be a measured response of the pathogen for maintaining its own Ca^2+^ homeostasis as well as rewire host's autophagic response for its survival. This maintenance of low intracellular and high extracellular calcium concentrations seems to be a conserved phenomenon in many bacteria (Rosch et al., 2008; Fujisawa et al., 2009; Faxén et al., 2011). Interplay and cross talk among various P-type ATPases through different stages of Mtb infection seems to be important in maintaining pathogen's ionic milieu, in response to stress conditions in the host.

Surge in calcium levels seen in macrophages after 1 and 2 h of infection with Mtb (Figure 4) and lack of such increase in ctpFCKD, indicates the importance of CtpF-mediated calcium flux from the pathogen to the host to modulate downstream events. The resultant higher calcium milieu in the macrophages may be responsible for negative regulation of *ctpE*, as seen earlier in transcriptomic survey of intracellular mycobacteria (Botella et al., 2011) and calcium mediated negative regulation of *ctpE* in *in vitro* cultures (Gupta et al., 2017). The lower calcium milieu in macrophages infected with ctpFCKD, could induce *ctpE*-mediated calcium uptake into Mtb from host intracellular stores, restoring the calcium levels after 4 h of infection (shown in Figure 4). Although our *ex vivo* experiments suggest dynamic calcium exchange between pathogen and the host in early time points of infection, further investigation of calcium traffic between Mtb and host at different stages of infection would aid in better understanding of features that regulate survival, persistence and clearance mechanisms of Mtb in the host.

In its engagement with the host, Mtb employs innumerable strategies for its uptake, survival in harsh environment, and subsequently, to proliferate or persist intracellularly. Inhibition of autophagy is one of the prime munitions of Mtb (Gutierrez et al., 2004; Castillo et al., 2012). Increase in Mtb replication seen in TB, as well as in TB-HIV co-infected human macrophages by autophagy inhibitors illustrate the point (Andersson et al., 2016). To impair host autophagic mechanisms, Mtb uses myriad of strategies, first being induction of an army of cytokines expression such as IL-4 (Ní Cheallaigh et al., 2011) and IL-13 (Jo, 2013). Another approach is modulation of mTOR, a major inhibitor of autophagic flux (Jo, 2013). Two independent mechanisms of mTOR modulation are understood so far. In the first, Eis-mediated acetylation by Mtb increases IL-10 expression and activates PI3K/Akt/mTOR/p70S6K pathway (Duan et al., 2016). In the second mechanism, Esx-1 mediated ESAT-6 release inhibits autophagy by activation of mTOR (Romagnoli et al., 2012; Dong et al., 2016). mTOR is regulated by calcium release from lysosomes, resulting in binding and activation of calmodulin (CAM). The latter, in turn, activates kinase activity of multi-protein mTORC1 complex comprising mTOR, mLST8, DEPTOR, Ttil/Tel2, PRAS40, and Raptor (Li et al., 2016; Saxton and Sabatini, 2017), leading to autophagy inhibition. Our results suggest that not only lysosomal calcium, but the bacterial calcium efflux may also act as an mTOR activator. By virtue of less calcium efflux in ctpFCKD, decrease in mTOR activation and concomitant increase in autophagy was observed. Our findings, that the organism engages a calcium ATPase for throwing out the ion, thereby altering cellular calcium levels to impact host signaling mechanisms, adds another layer to its multi-layered autophagy inhibition strategy for intracellular survival.

That the CtpF or its homologs are conserved across different life forms, highlights the importance of the regulation of calcium ion concentration across the species but undervalues its credentials as a potential drug target in Mtb. Nevertheless, subtle variation in its structure, kinetic properties and characteristics in different organisms may result in differences in calcium transport. Notably, the observation that CtpF is not essential *in vitro* but provides a survival advantage to the intracellular Mtb, reaffirms its potential as a drug target. The modeling studies presented by us and others (Santos et al., 2020) provide some insights in this direction. Similar to SERCA ATPase (Olesen et al., 2007; Di Marino et al., 2015), CtpF may also exist in a number of conformational states including the calcium bound and unbound forms. The inhibitor of the ATPase, C PA appears to lock the protein in calcium free conformation similar to its action on SERCA (Santos et al., 2020). The binding sites for the drug and calcium are largely non-overlapping and the inhibition seen (Santos et al., 2020) is likely by allosteric mode and the drug binding would block the channel of calcium entry. Thus, binding of the drug and calcium to the protein appears to be mutually exclusive. The points underscore the potential of developing CPA derivatives as well as new molecules to target CtpF to impact calcium traffic in intracellular Mtb.

## Materials and Methods

### Bacterial Strains

Mtb H37Rv and *M. smegmatis* strains were grown either on Middlebrook 7H11 agar solid media or in 7H9 broth (Difco, USA) with or without oleic acid-albumin-dextrose-catalase (OADC) enrichment. Hygromycin and kanamycin were used at a concentration of 50 and 25 μg ml^−1^, respectively, for culturing Mtb dcas9-pRH2521 and ctpFCKD strains. For culturing recombinant *E. coli* DH5α strains, 150 and 50 μg ml^−1^ hygromycin and kanamycin were used, respectively.

### Generation of *M. smegmatis* and Mtb Strains

The coding DNA sequence (CDS) of *ctpF* was cloned in acetamide-inducible vector pMyNT at BamHI and HindIII sites using ctpFpMyNT FP and ctpFpMyNT RP primers (Table S1). pMyNT vector was a gift from Annabel Parret & Matthias Wilmanns (Addgene plasmid # 42191; RRID: Addgene_42191). The positive clones were confirmed using restriction digestion and Sanger's sequencing. The recombinant plasmid was transformed into *M. smegmatis* mc^2^155, induced with 2% acetamide at 0.4 OD for 12 h at 37°C and expression of CtpF was confirmed using anti-his antibody. The resultant strain was called MsCtpF. *ctpF* conditional knockdown (ctpFCKD) was generated in Mtb by CRISPR-Cas9 approach (Singh et al., 2016). Two small guide RNAs (sgRNAs) targeting the non-template strand of *ctpF* gene: sg1 (319 bp downstream of translational start site) and sg2 (408 bp downstream of translational start site) were cloned in pRH2521 vector and transformed in Mtb H37Rv-dCas9 to generate two separate strains. The colonies obtained after transformation of both the sgRNAs were cultured and screened for decrease in expression of *ctpF* transcript, upon sgRNA induction with 600 ng/ml ATc.

### Preparation of Membrane Vesicles

The MsCtpF culture was induced with 2% acetamide at 0.4 OD for 12 h at 37°C. The membrane fraction was isolated from the induced culture as described previously (Rezwan et al., 2007). The membrane pellet was washed and solubilized in buffer containing 50 mM Tris pH 7.4, 100 mM NaCl, 10% glycerol, containing DDM (0.1 g per g of membrane) and the suspension was centrifuged at 16,000 g for 10 min and the supernatant contained membrane vesicles. The membrane vesicles thus obtained were stored at −80°C until further use.

### ATPase Assay

The ATPase activity was determined by measuring the release of inorganic phosphate (Pi) as described previously with some modifications (Padilla-Benavides et al., 2013). The reaction mixture consisted of assay buffer (60 mM Tris pH 7.4 and 100 mM KCl), 1 mM ATP and 4 μg of membrane vesicles from MspMyNT and MsCtpF strains. The reactions were carried out with 0.2–3 mM of KCl, NaCl, MgCl_2_, and CaCl_2_. The effective Pi release was calculated by normalizing Pi release at all the concentrations of metal ion with respect to no metal ion and only vector reading. The metal ion stimulated activity was measured by plotting Pi release vs. metal concentration and the curves were fit to Michaelis-Menten equation (Non-linear regression) to obtain Km and Vmax values. To assess the effect of chelators, the reactions were supplemented with different concentrations of EDTA and EGTA in separate sets. To check the inhibition of ATPase activity, the reactions were supplemented with different concentrations of sodium orthovanadate.

### Analysis of Growth

Primary cultures of Ms, MspMyNT and MsCtpF strains were used for inoculating 7H9 media with an initial OD of 0.02. The cultures were induced with 2% acetamide and growth curve was set in honeycomb well plate in Bioscreen C Automated Microbiology Growth Curve Analysis System. The OD_600_ values were recorded after 2 h till 48 h and plotted in Graph Pad prism 6.0 software. To see growth rescue of MsCtpF, the media was supplemented with 1–10 mM CaCl_2_ and 1–10 mM MgCl_2_ in separate plates. For Mtb H37Rv growth curves, secondary cultures were inoculated at an initial OD of 0.02 in 7H9-ADC medium and OD was recorded at every 48 h for 12 days.

### Biofilm, Pellicle Formation, and Sliding Motility

Biofilm assay for secondary induced cultures of Ms, MspMyNT and MsctpF strains was set up in a 12 well plate with/without 2 mM CaCl_2_ and MgCl_2_. The plate was kept at 37°C for 7 days for biofilm formation. For pellicle formation, cultures were left undisturbed in tubes at 37°C for 7 days. Sliding motility assay was carried out as described (Martínez et al., 1999). Briefly, 1 μl of induced cultures were spotted on 7H9 agarose plates containing 0.47 g/100 ml 7H9 powder, 0.5% tryptone, 0.2% glycerol, 0.3% agarose, 2% acetamide, with/without 2 mM CaCl_2_ and MgCl_2._ The plates were incubated at 37°C for 3 days.

### Homology Modeling of CtpF

Homology model of CtpF was generated using MODELLER9.21 package (Webb and Sali, 2016) using the structure of Ca^2+^-bound SERCA1a (PDB code 1SU4). The sequence of CtpF was aligned with the SERCA1a structure using align2d() command in MODELLER9.21 generating structure based target-template sequence alignment. Target-template alignment and 1SU4 template structure was used to construct five models of CtpF using model-single pyscript in MODELLER9.21. The best model with the lowest value of the MODELER objective function, the DOPE assessment score (−95859.38281), or the highest GA341 assessment score (1.00000) was selected for analysis. Analysis of structures including structural superposition and generation of molecular graphics was performed using PyMOL (DeLano, 2002).

### Multiple Sequence Alignment

Two multiple sequence alignments: CtpF with well-studied Ca^2+^-ATPases from rabbit, pig, human and bovine and CtpF with orthologs from Mtb complex and representative species of actinomycetes, were generated using Clustal Omega service (Madeira et al., 2019). For analysis, the generated alignment was rendered to represent residue conservation and similarities along with secondary structure information from aligned sequences using program ESPript 3.0 (Robert and Gouet, 2014).

### THP-1 Macrophage Infection

THP-1 cells were seeded at cell density of 10^4^/well in a 96 well plate containing RPMI 1640 complete medium and activated using 20 ng/ml PMA for 16 h. The media containing PMA was aspirated and the cells were incubated with incomplete medium without antibiotics. The cells were infected with different Mtb strains at MOI of 1:10 for 4 h, extracellular bacteria were killed using 40 ug/ml gentamicin for 45 min, and intracellular bacteria were harvested at 0, 24, 48 h. Serial dilutions were prepared, plated on 7H11-OADC plates and CFU was calculated after 3 weeks of incubation.

### Measurement and Imaging of Intracellular Calcium in Macrophages

The Mtb H37Rv, dcas9-pRH2521, ctpFCKD cultures were induced during exponential phase and used to infect peritoneal macrophages at MOI of 1:10. After 1, 2, and 4 h of infection, extracellular bacteria were removed by washing and the cells were stained with Fura Red, AM dye (Thermo Fisher Scientific, USA) for 1 h at 37°C, followed by 20 min incubation at room temperature (Rohrbach et al., 2005). The fluorescence values were measured at two different excitation-emission wavelengths: 435/630 nm and 470/650 nm and their ratio were taken. In a separate experiment, the cells were infected and stained with Fura Red, AM dye, counter-stained with 10 ug/ml DAPI and mounted on a glass slide with prolong glass antifade (Thermo Fisher Scientific, USA). As a positive control, the infected cells after the indicated time points were incubated with BAPTA, AM (Thermo Fisher Scientific, USA) for 30 min to chelate the calcium and then loaded with Fura Red, AM dye. The macrophages were visualized under 63X objective, using Zeiss LSM-880 microscope with excitation/emission maxima wavelengths: 488/660 nm and the images were pseudo-colored in green. Post-acquisition processing of images was done using Zen blue 2.3 lite software.

### Quantitative Real-Time PCR

Total RNA was extracted from Mtb H37Rv using FastRNA® Pro Blue Kit (MP Biomedicals, USA) according to manufacturer's instructions. RNA was treated with Turbo DNase I (Thermo Fisher scientific, USA) to remove genomic DNA contamination. RNA was quantified and cDNA was prepared using High capacity cDNA reverse Transcriptase kit (Thermo Fisher scientific, USA). Gene expression levels were determined by quantitative real time PCR using cDNA with DyNAmo Color Flash SYBR Green Master Mix (Thermo Fisher scientific, USA) on CFX384 Touch Real-Time PCR Detection System (Bio-Rad, USA). 16s rRNA was used as an internal control and relative expression levels were calculated using 2^−ΔΔ^Ct method as described previously (Schmittgen and Livak, 2008). The primer sequences are listed in Table S1. Nitric oxide stress was given to Mtb according to a method described previously (Voskuil et al., 2003). Mtb was exposed to hypoxia and acidic media as described earlier (Rohde et al., 2007; Garg et al., 2015).

### Isolation of Murine Peritoneal Macrophages

Four to six weeks old, male and female BALB/c mice were procured from The Jackson Laboratory and maintained at the Central Animal Facility (CAF), Indian Institute of Science (IISc). Eight percent Brewer's thioglycollate was injected in the peritoneal cavity of mice. Four days post-injection, the peritoneal exudates containing elicited macrophages were harvested in ice-cold 1X PBS. The cells were maintained in cell culture dishes containing Dulbecco's Modified Eagle Medium (DMEM) supplemented with 10% heat-inactivated Fetal Bovine Serum (Gibco, Thermo Fisher Scientific, USA) at 37°C in 5% CO_2_ incubator.

### *Ex vivo* Infection With Mtb and Immunoblotting

Equal number of the peritoneal macrophages were seeded in 24-well plate and infected with different Mtb strains at an MOI of 1:10. At different time points, cells were washed and lysed in RIPA buffer [50 mM Tris-HCl (pH 7.4), 1% NP-40, 0.25% sodium deoxycholate, 150 mM NaCl, 1 mM EDTA, 1 mM PMSF, 1 μg/ml each of aprotinin, leupeptin, pepstatin, 1 mM Na_3_VO_4_, 1 mM NaF] on ice for 30 min and total protein was collected. An equal amount of protein from each cell lysate was electrophoresed on SDS-PAG and transferred onto PVDF membrane (Millipore, USA) by semi-dry Western blotting method (Bio-Rad, USA). Non-specific binding was blocked with 5% non-fat dry milk powder in TBST [20 mM Tris-HCl (pH 7.4), 137 mM NaCl, and 0.1% Tween 20] and incubated overnight at 4°C with primary antibody diluted in TBST with 5% BSA. The blots were washed and incubated with anti-rabbit or anti-mouse secondary-HRP conjugated antibodies for 2 h. The blots were developed using enhanced chemiluminescence detection system (Perkin Elmer, USA) as per manufacturer's instructions. β-ACTIN was used as a loading control. Phospho-mTOR (Ser2448) and HRP-conjugated anti-rabbit IgG antibodies were from Cell Signaling Technology and Jackson Immuno Research, USA, respectively. Anti-β-ACTIN-Peroxidase and anti-LC3B antibodies were from Sigma-Aldrich, USA.

### Statistical Analyses

Quantitative data are expressed as mean ± s.d. from three independent experiments. Statistical analyses were performed using GraphPad Prism 6.0. To determine the statistically significant differences between experimental groups, an unpaired Student's *t-*test was performed.

## Data Availability Statement

All datasets presented in this study are included in the article/Supplementary Material.

## Ethics Statement

The protocols for animal experiments were approved by national guidelines of the Committee for the Purpose of Control and Supervision of Experiments on Animals (CPCSEA), Government of India. All experiments with Mtb H37Rv and mice were approved by Institutional Animal Ethics Committee and Institutional Biosafety Committee of Indian Institute of Science, Bangalore, India. The animal study was reviewed and approved by Institutional Animal Ethics Committee of Indian Institute of Science, Bangalore, India.

## Author Contributions

RG and VN conceived the idea, designed experiments, and wrote the manuscript. RG and SMB performed Mtb H37Rv experiments. HB performed bioinformatic analysis. PS, SR, and RV performed experiments with *M. smegmatis* strains. KNB supervised autophagy and signaling experiments. All authors contributed to the article and approved the submitted version.

## Conflict of Interest

The authors declare that the research was conducted in the absence of any commercial or financial relationships that could be construed as a potential conflict of interest.

## Supplementary Material

Supplementary Figure 1 Homology model of CtpF. **(A)** Overall topology. The model is colored in rainbow with colors changing gradually from the N-terminus (blue) to the C-terminus (red). **(B)** Domain colored model of CtpF. Domain A (smudge), actuator or anchor; Domain N (yellow), nucleotide; Domain P (dark salmon), phosphorylation, Domain T (blue), transport, and Domain S (cyan), class specific support domain, are shown. D333 is the proposed residue of phosphorylation in P domain in CtpF, based on its structural conservation with the phosphorylation residue D351 in P domain in SERCA1a (PDB code 1SU4). **(C)** Ca^2+^ binding sites mapped in homology model of CtpF. Ca^2+^ ions are depicted as magenta spheres. Amino acid residues are shown in a stick model and are colored as green for CtpF and yellow for SERCA1a. Amino acid residues are numbered as residue (CtpF/SERCA1a) or residue (CtpF)/residue (SERCA1a). **(D)** The table shows transmembrane helix position for the calcium binding residues in CtpF and SERCA1a.

Supplementary Figure 2 The multiple sequence alignment of CtpF and CtpF orthologs across different *actinomycetes* spp. **(A)** and with calcium ATPases with known structures **(B)**. **(A)** The alignment of CtpF protein sequences from different species of MTBC complex (*M. africanum, M. bovis, M.canetti, M. microti*), *M. smegmatis*, and other actinomycetes (*Streptomyces* and *Burkholderia* sp.). **(B)** The alignment of CtpF protein sequence from Mtb, ATP2A2 from *Sus scrofa* (Pig), ATP2A2 from *Homo sapiens* (Human), ATP2A1 from *Oryctolagus cuniculus* (Rabbit) and ATP2A1 from *Bos taurus* (Bovine). Conserved residues are marked in white on a red background while similar residues are in red. Residues in blue frame depict similarity across groups. Residues that bind adenosine moiety of ATP in the N domain, Ca^2+^ in the transmembrane region and the phosphorylation residue in the P domain as inferred from structural and biochemical studies of SERCA from rabbit, pig, human and bovine, are indicated by yellow circle, blue squares, and green stars, respectively, on the alignment. Secondary structural elements and residue numbering indicated above the alignment are extracted from the CtpF model.

Supplementary Figure 3 MsCtpF shows changes in cell envelope. **(A)** Pellicle assay for acetamide induced Ms, MsPMyNT, MsCtpF cultures in 7H9 media (upper panel); 7H9 media supplemented with 2 mM CaCl_2_ (middle panel) and 2 mM MgCl_2_ (lower panel). **(B)** Biofilm assay without (upper panel)/ with 2 mM CaCl_2_ (middle panel) and 2 mM MgCl_2_ (lower panel). **(C)** Sliding motility assay without (upper panel)/ with 2 mM CaCl_2_ (middle panel) and 2 mM MgCl_2_ (lower panel). The experiment was repeated thrice, and the result shown is the representative image.

## Abbreviations

CtpF: Cation transporting P-type ATPase F
Mtb: *Mycobacterium tuberculosis*
CKD: conditional knockdown
mTOR: mammalian target of rapamycin
CRISPR: Clustered Regularly Interspaced Short Palindromic Repeats.
