# Supplementary material for: Mycobacterium tuberculosis Calcium Pump CtpF Modulates the Autophagosome in an mTOR-Dependent Manner
Source: Front Cell Infect Microbiol. 2020 Sep 16;10:461. doi: 10.3389/fcimb.2020.00461 (PMC7525011; doi:10.3389/fcimb.2020.00461)
Supplement: Supplementary file 1 [file Data_Sheet_1.ZIP › table S1.docx]

**Table S1:** List of oligonucleotides used in the study

| **Primer name** | **Forward primer (5'→3')** | **Reverse primer (5'→3')** |
| --- | --- | --- |
| ctpF sgRNA1 | GGGACTGCAGTGCGGCCTCTGCCT | AAACAGGCAGAGGCCGCACTGCAG |
| ctpF sgRNA2 | GGGACCGGGAACCAGCTCTTCGGA | AAACTCCGAAGAGCTGGTTCCCGG |
| ctpFpMYNT | GCGCGGATCCGATGTCGGCGTCAGTGTCT | GCGCAAGCTTTCATGGCGGTTGCGCCCG |
| ctpF RT | TCGCTGATCGTCGCAATATC | AATCTCACCGAGTTCGGTTTC |
| 16s RT | ACGCGAAGAACCTTACCTGG | CCCAACATCTCACGACACGA |
