# Supplementary figures and images for: Mycobacterium tuberculosis Calcium Pump CtpF Modulates the Autophagosome in an mTOR-Dependent Manner
Source: Front Cell Infect Microbiol. 2020 Sep 16;10:461. doi: 10.3389/fcimb.2020.00461 (PMC7525011; doi:10.3389/fcimb.2020.00461)

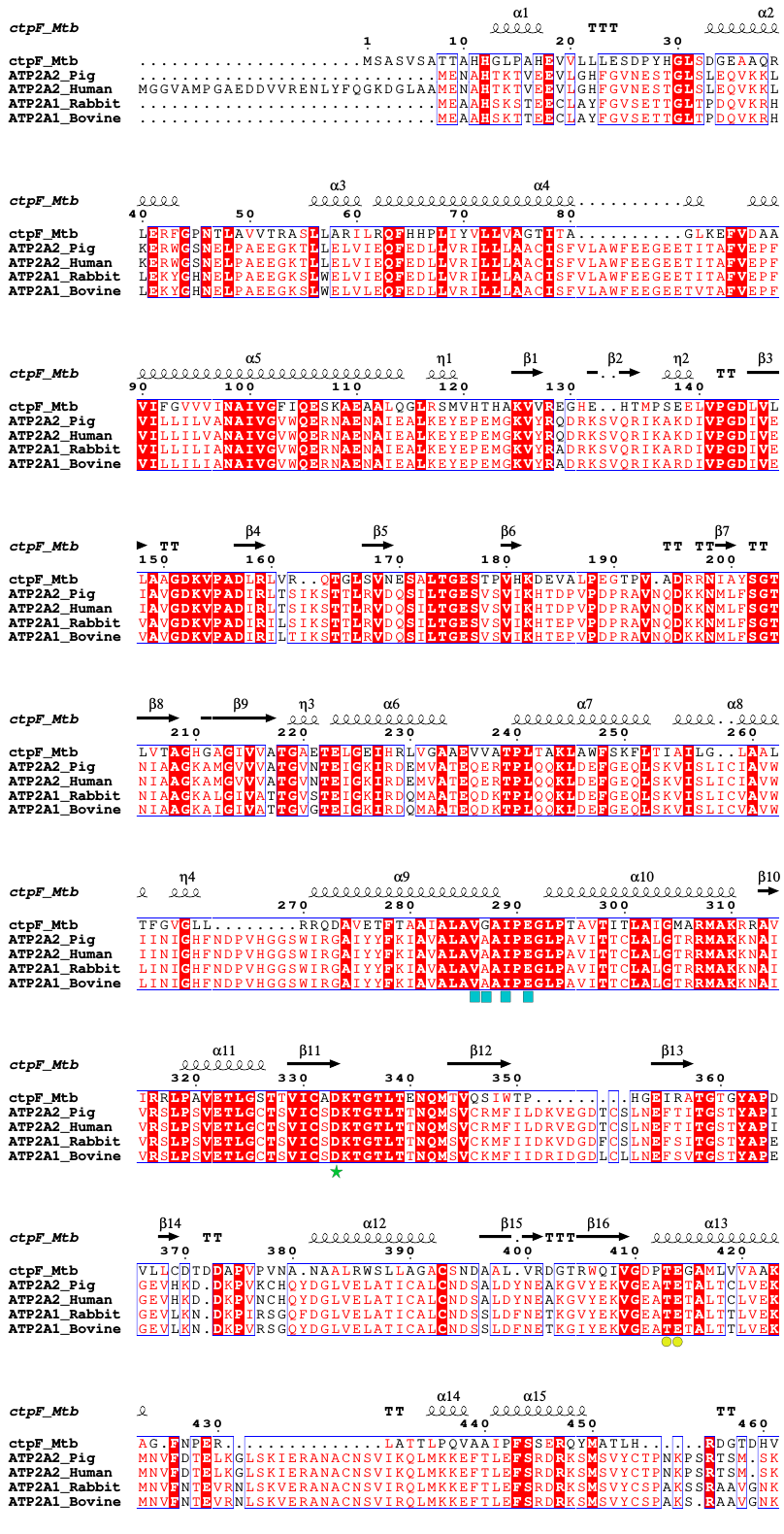

Supplement: Supplementary file 1 [file Data_Sheet_1.ZIP › Fig S2B-1.tif]

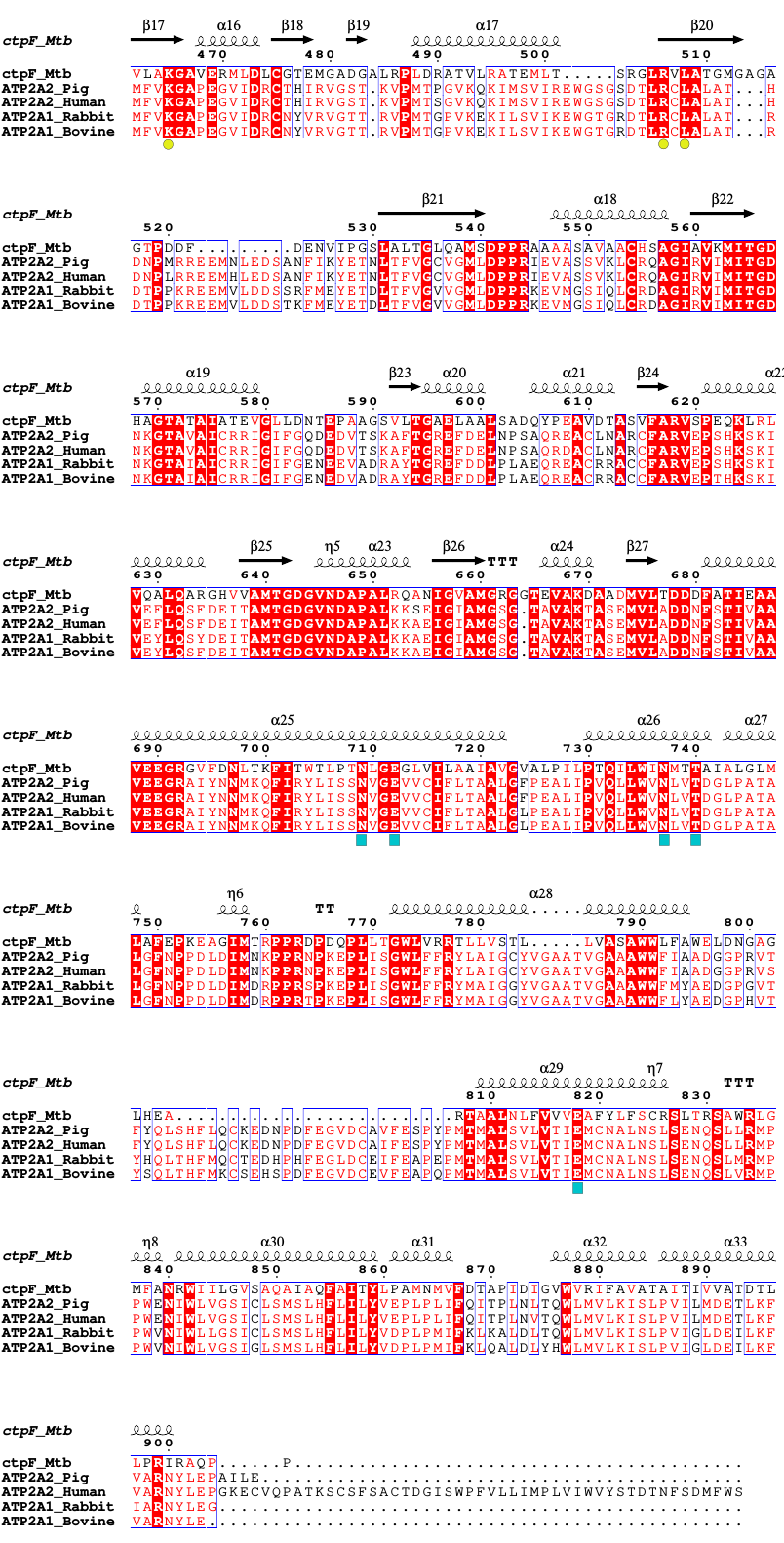

Supplement: Supplementary file 1 [file Data_Sheet_1.ZIP › Fig S2B-2.tif]

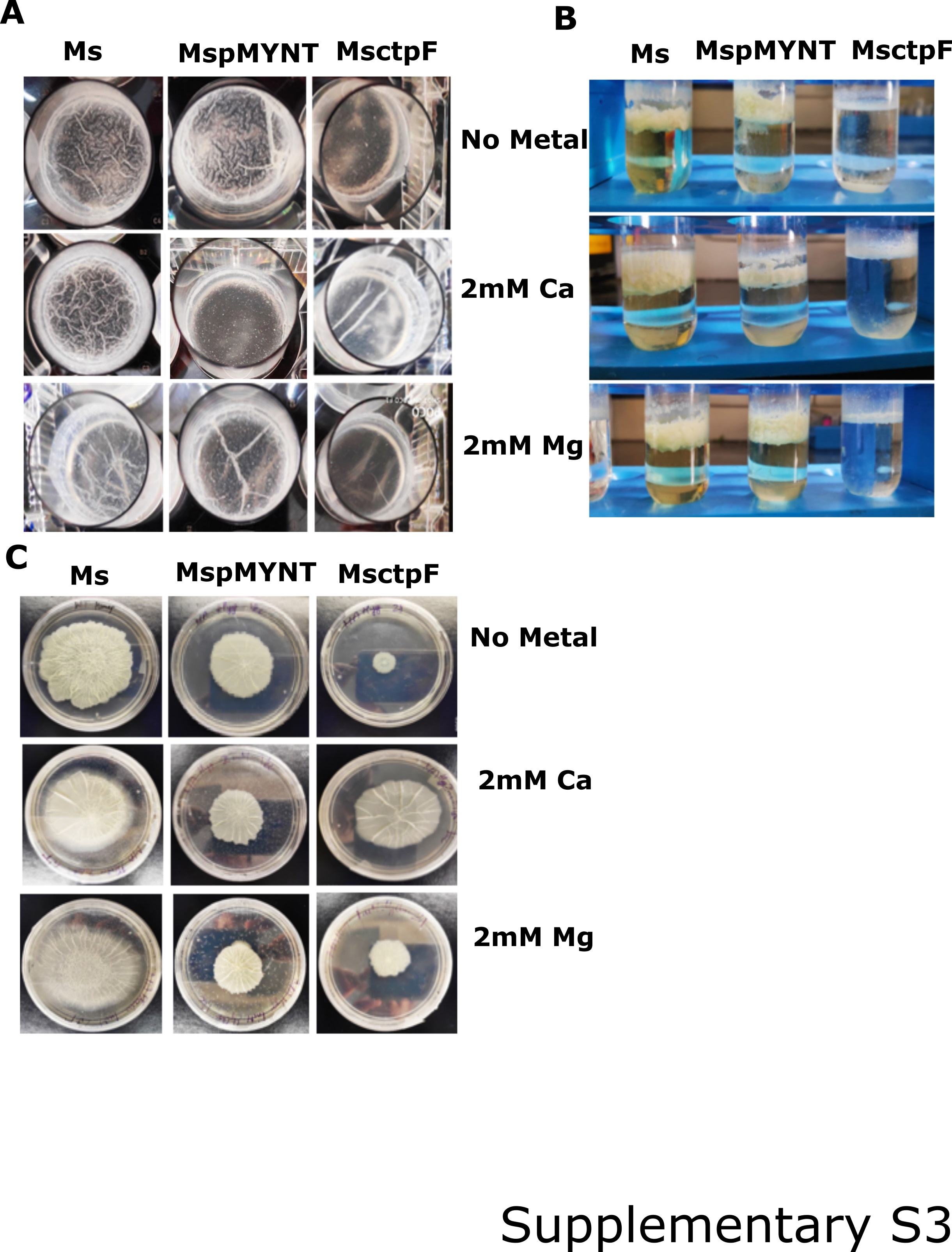

Supplement: Supplementary file 1 [file Data_Sheet_1.ZIP › Fig S3.jpeg]

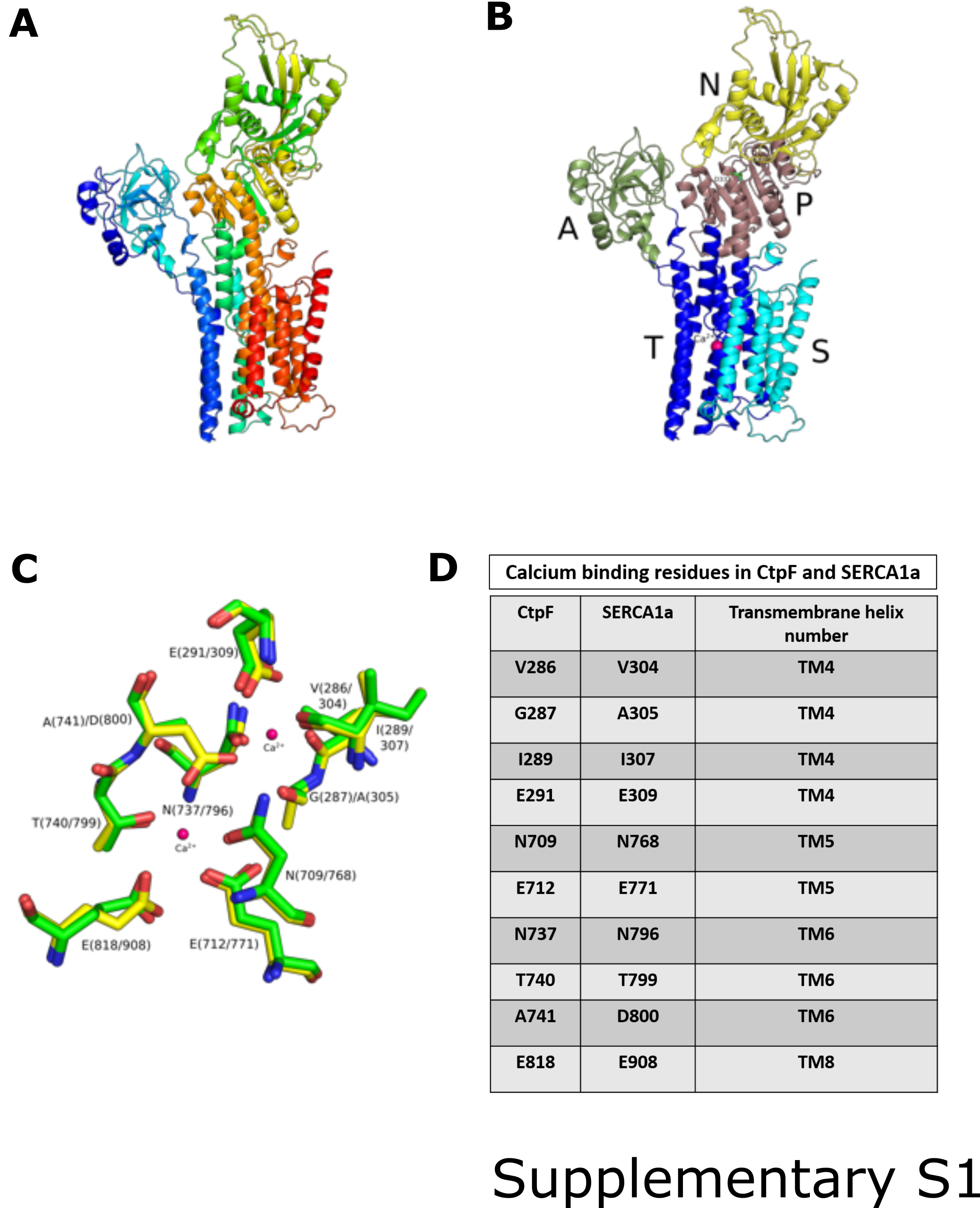

Supplement: Supplementary file 1 [file Data_Sheet_1.ZIP › Fig S1.tif]

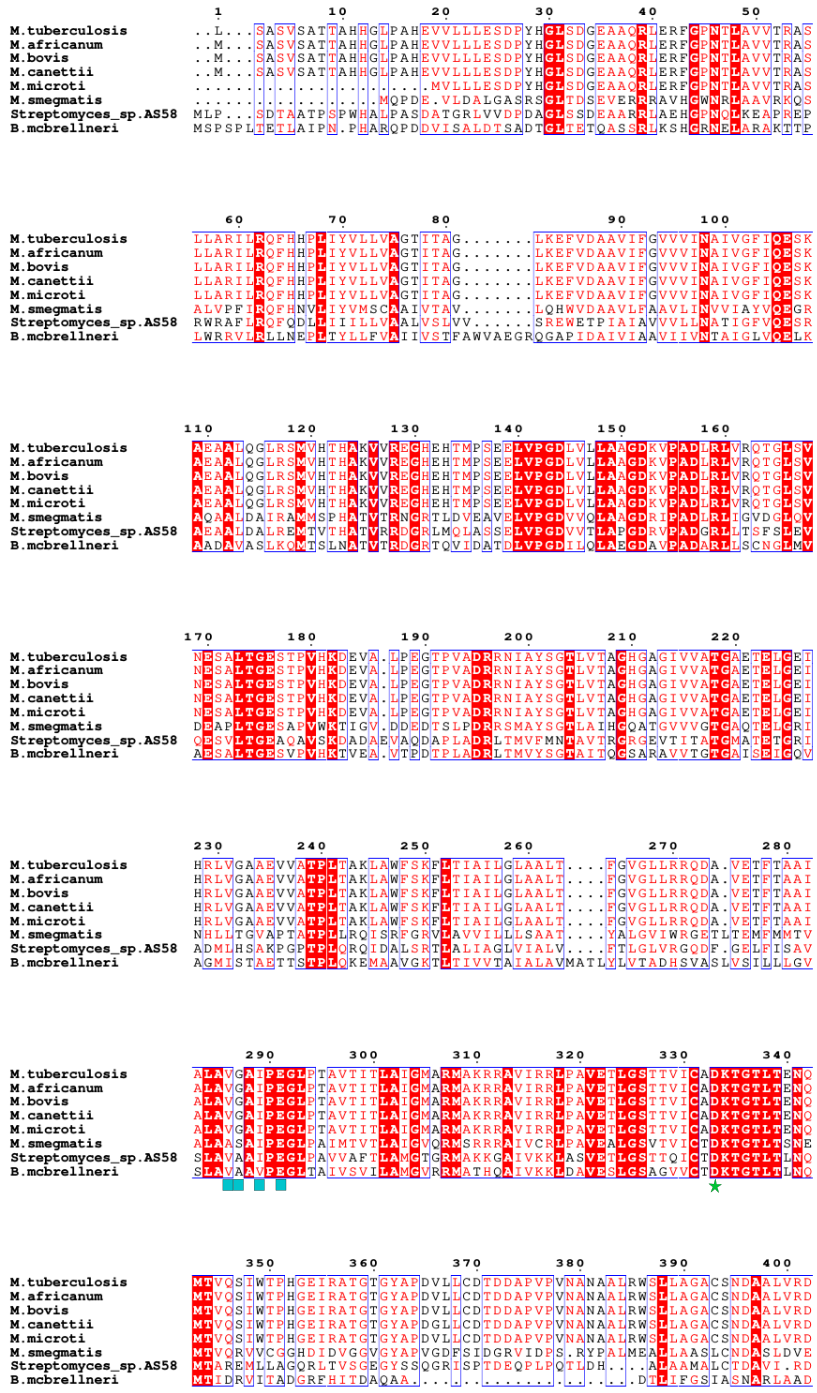

Supplement: Supplementary file 1 [file Data_Sheet_1.ZIP › Fig S2A-1.tif]

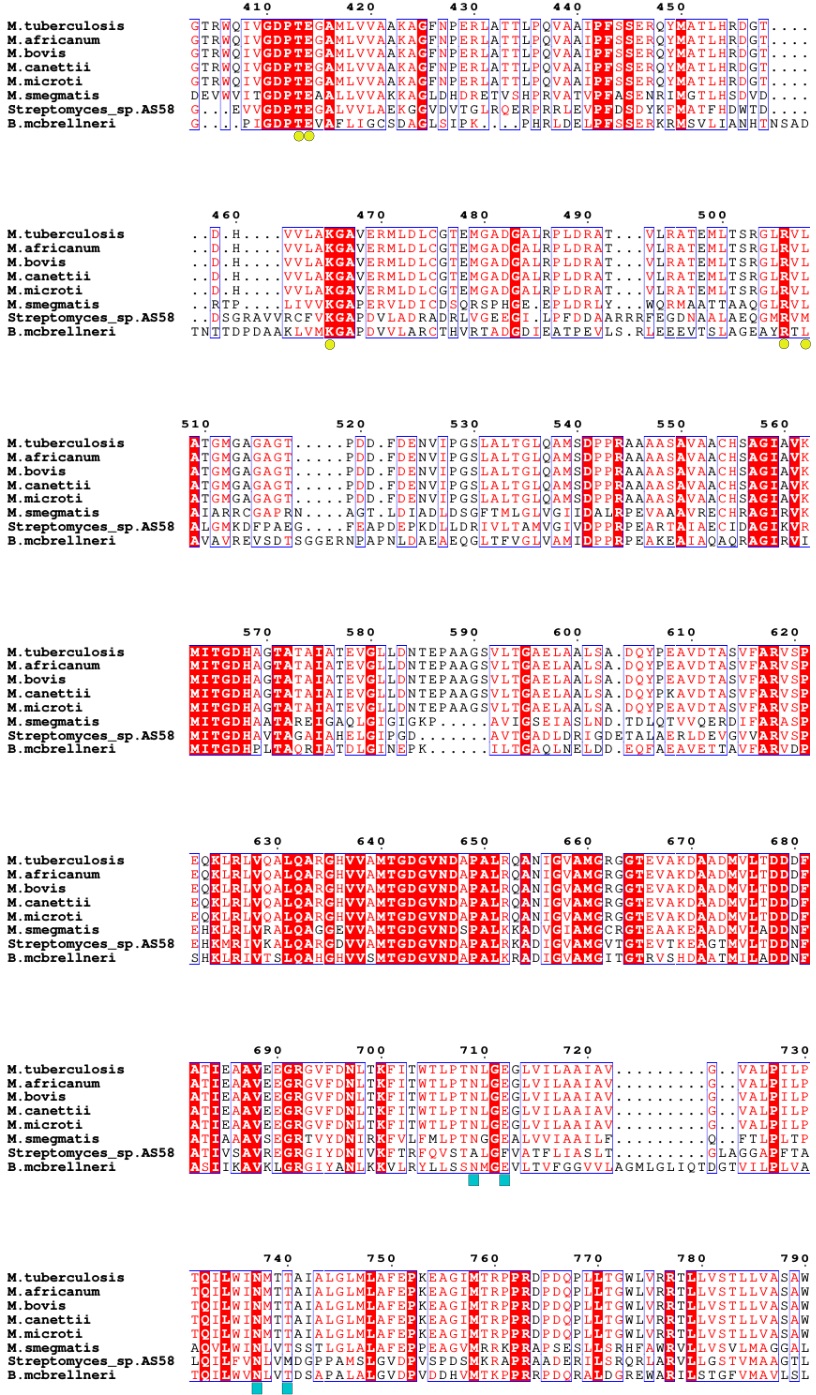

Supplement: Supplementary file 1 [file Data_Sheet_1.ZIP › Fig S2A-2.jpg]

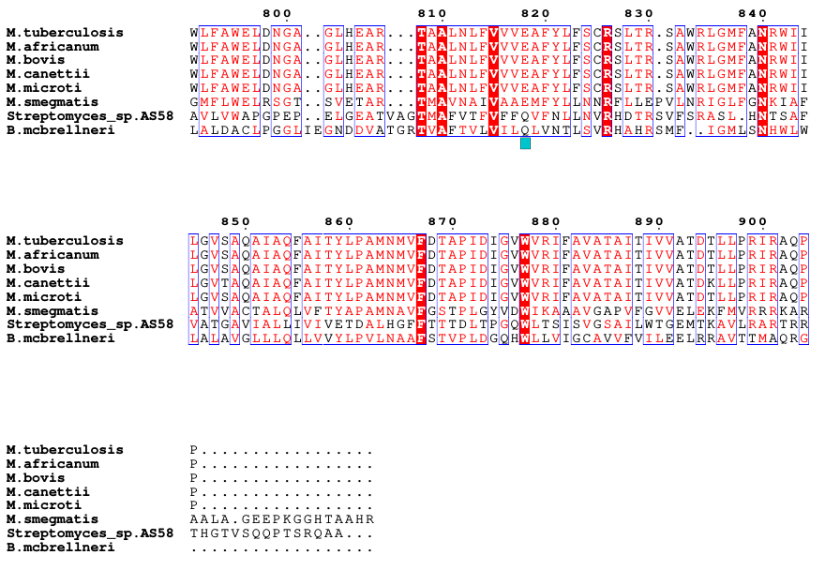

Supplement: Supplementary file 1 [file Data_Sheet_1.ZIP › Fig S2A-3.tif]
